# Pregnancy-Associated Atypical Hemolytic Uremic Syndrome With Kidney Recovery Despite Delayed Initiation of Complement Blockade

**DOI:** 10.7759/cureus.108064

**Published:** 2026-04-30

**Authors:** Abdurrahman Hamadah, Tarek Eleraky, Dileep Kumar, Dhanya Mohan, Aisha Bibi, Herlitz Leal, Amna Alhadari

**Affiliations:** 1 Nephrology, Dubai Academic Health Corporation (DAHC), Dubai, ARE; 2 Nephrology, Dubai Hospital, Dubai Academic Health Corporation (DAHC), Dubai, ARE; 3 Pathology, Cleveland Clinic, Cleveland, USA

**Keywords:** acute kidney injury, atypical hemolytic uremic syndrome (ahus), complement, hemolytic uremic syndrome (hus), kidney failure, pregnancy

## Abstract

Atypical hemolytic uremic syndrome (aHUS) is a complement-mediated thrombotic microangiopathy (TMA) characterized by microangiopathic hemolytic anemia, thrombocytopenia, and acute kidney injury, and pregnancy is a recognized trigger, particularly in the third trimester and postpartum period. We report a 29-year-old primigravida at 33 weeks’ gestation who presented with severe acute kidney injury (AKI), thrombocytopenia, anemia, elevated lactate dehydrogenase, and hyperbilirubinemia with otherwise normal liver enzymes. The ADAMTS13 activity was normal, and autoimmune serologies were negative. She required urgent hemodialysis and underwent emergency cesarean section for maternal indications; however, renal failure persisted postpartum with dialysis dependence.

Kidney biopsy demonstrated thrombotic microangiopathy with arteriolar fibrin thrombi. Genetic testing identified a heterozygous pathogenic splice-site variant in complement factor H, supporting the diagnosis of complement-mediated aHUS. Complement inhibition with eculizumab was initiated approximately five weeks after presentation and later transitioned to ravulizumab. Despite delayed therapy and initial dialysis dependence, she achieved dialysis independence five weeks after initiation of complement blockade, with partial renal recovery and normalization of platelet count. This case emphasizes the need to consider complement-mediated TMA in pregnant patients with severe renal dysfunction disproportionate to liver enzyme abnormalities and demonstrates that meaningful renal recovery remains possible even when dialysis is required, and complement inhibition is not initiated immediately.

## Introduction

Atypical hemolytic uremic syndrome (aHUS) is a rare, life-threatening thrombotic microangiopathy (TMA) driven by dysregulation of the alternative complement pathway. It is clinically defined by the triad of microangiopathic hemolytic anemia (MAHA), thrombocytopenia, and acute kidney injury (AKI) [1,2]. Unlike Shiga toxin-associated hemolytic uremic syndrome (HUS), aHUS frequently arises from inherited or acquired defects in complement regulatory proteins, including complement factor H (CFH), factor I, membrane cofactor protein (CD46), and C3 [2-4]. Pregnancy is a well-recognized trigger for complement-mediated TMA, typically occurring in the third trimester or postpartum period [3-5]. Differentiating aHUS from preeclampsia and hemolysis, elevated liver enzymes, and low platelets (HELLP) syndrome remains challenging because of overlapping clinical features. However, severe kidney dysfunction out of proportion to liver enzyme abnormalities and persistence of kidney injury after delivery should raise suspicion for complement-mediated TMA [5].

Eculizumab, a monoclonal antibody targeting complement protein C5, has significantly improved outcomes in aHUS [6]. Although early initiation is associated with better renal recovery, delayed therapy does not necessarily preclude improvement [7]. We describe a case of third-trimester pregnancy-triggered aHUS associated with a pathogenic CFH splice-site variant, with dialysis dependence at presentation and subsequent renal recovery following delayed complement inhibition.

## Case presentation

**Figure 1:**
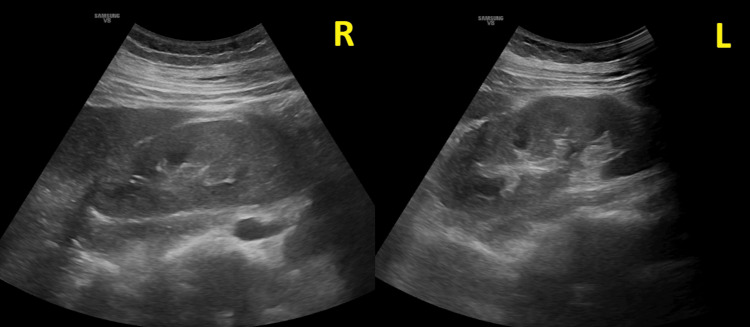
Ultrasound of right (R) and left (L) kidneys

A 29-year-old primigravida with obesity but otherwise healthy was followed regularly from 16 weeks gestation by her primary provider. She had no prior history of hypertension, diabetes, or kidney disease. At 28 weeks gestation, she developed generalized pruritus, predominantly affecting the palms and soles. Blood work at that time was unremarkable, including liver function tests, except she was noted to have a mildly decreased platelet count at 121 × 10^9/L. The patient was followed closely, and at 33 weeks gestation, she presented with facial edema and elevated blood pressure (147/80 mmHg). Her BMI was 33.5 kg/m². She denied having a headache, visual disturbances, or epigastric pain. Fetal assessment was reassuring. At that time, she was noted to have a hemoglobin of 7.8 g/dL, platelets of 61 × 10^9/L, and creatinine of 6.43 mg/dL. Urinalysis showed 2+ protein and the presence of 5-10 RBCs/hpf on microscopy. Peripheral smear showed anemia with occasional fragmented red cells/schistocytes and thrombocytopenia. The ADAMTS13 activity was normal. She had an extensive workup for her presentation, which included anti-nuclear antibody (ANA), anti-dsDNA, extractable nuclear antigen panel, and cryoglobulins, which were negative. Renal ultrasound demonstrated normal-sized kidneys with increased cortical echogenicity (Figure 1). The main investigations are shown in Table 1. 

**Table 1:** Laboratory findings on presentation and initial investigation

Parameter	Patient value	Reference range
Hemoglobin	7.8 g/dL	12.0-15.0 g/dL
Hematocrit	23.2%	36-46 %
Platelets	61 ×10^9/L	150-410 ×10^9/L
WBC	4.9 ×10^9/L	4.0-11.0 ×10^9/L
Creatinine	6.43 mg/dL	0.5-0.9 mg/dL
Urea	153 mg/dL	12-40 mg/dL
Potassium	5.9 mmol/L	3.4-4.5 mmol/L
Sodium	132 mmol/L	136-145 mmol/L
Bicarbonate (HCO₃⁻)	15.1 mmol/L	20-28 mmol/L
Serum albumin	2.4 g/dL	3.9-5 g/dL
Lactate dehydrogenase (LDH)	678 U/L	105-222 U/L
Total bilirubin	3.72 mg/dL	0.2-1.2 mg/dL
Alanine aminotransferase (ALT)	17 U/L	<35 U/L
Aspartate aminotransferase (AST)	29 U/L	<35 U/L
Complement C3	0.83	0.9-1.8 g/L
Complement C4	0.39	0.1-0.4 g/L
ADAMTS13 activity	Normal	>10% activity
Urine protein/creatinine ratio	6.1 g/g	<0.15 g/g

**Figure 2:**
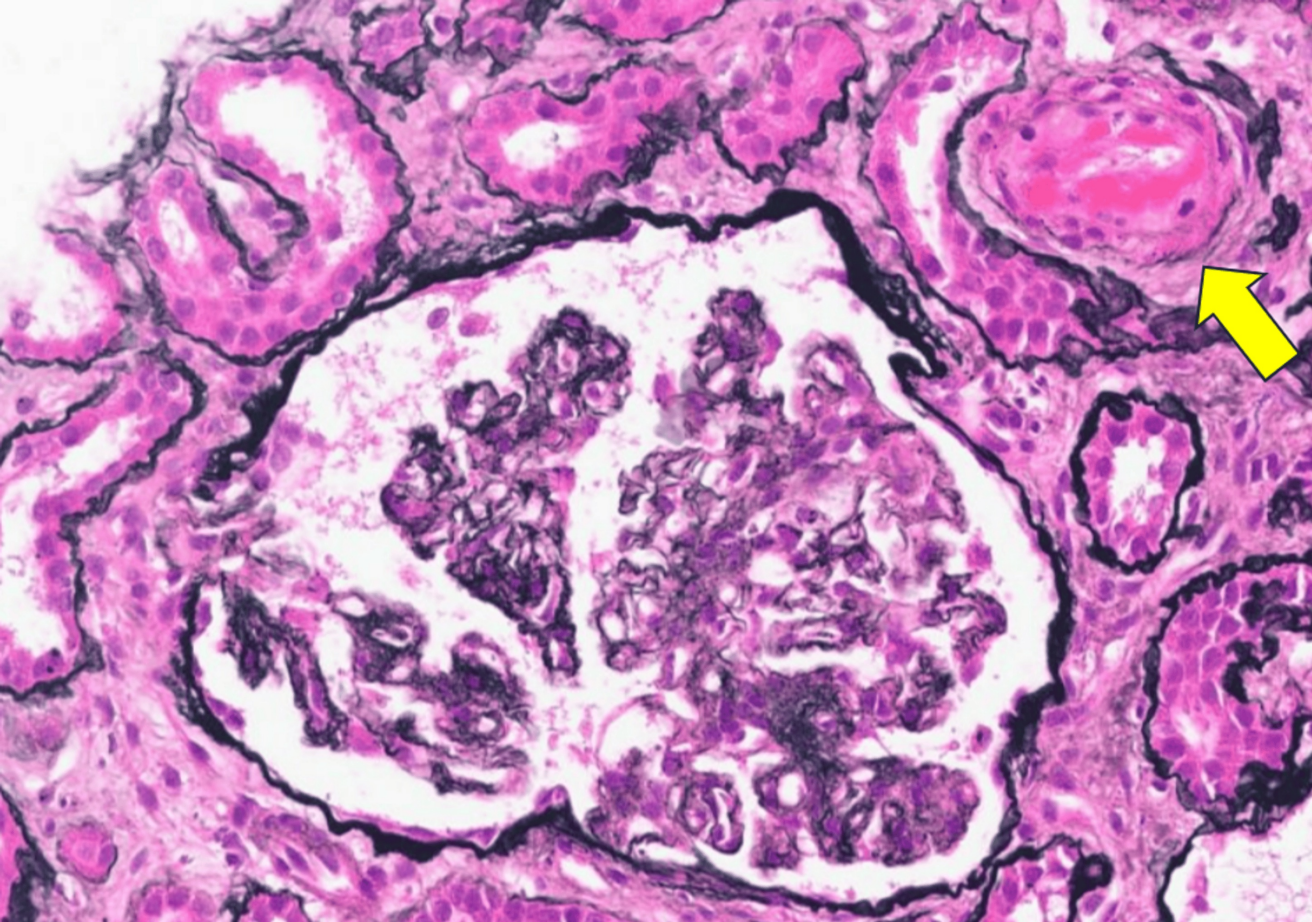
Ischemic glomerular changes and arteriole (arrow) showing an intraluminal fibrin thrombus (light microscopy, silver stain)

**Figure 3:**
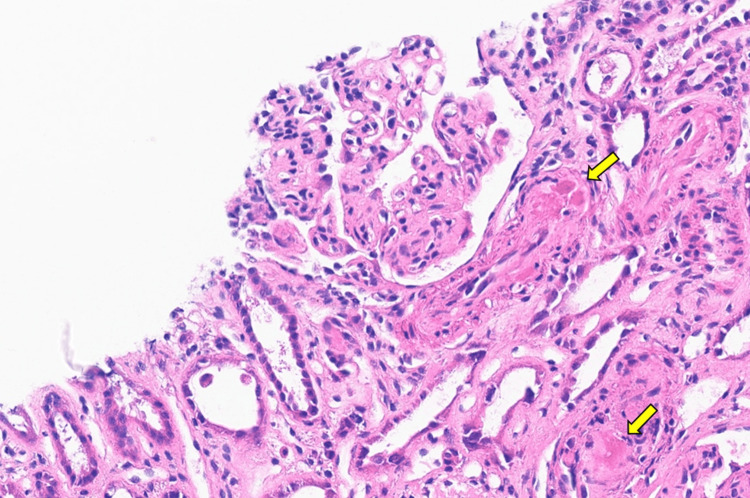
Arterioles with fibrin thrombi (arrows) and glomerulus with mesangiolysis (light microscopy, hematoxylin and eosin (H&E) stain)

The patient had progressive AKI and became anuric, and hemodialysis was initiated within 24 hours of presentation. She subsequently underwent an emergency cesarean section at 33 weeks of gestation, which was uneventful with the birth of a healthy 1950 gm male infant. Over the ensuing days postpartum, she showed no evidence of kidney recovery and remained hemodialysis dependent. Her hemolysis parameters, however, improved, and her platelets and other hemolysis parameters (LDH, haptoglobin, and bilirubin) trended towards normal. She subsequently underwent a kidney biopsy, which showed thrombotic microangiopathy predominantly in arterioles, with fresh fibrin thrombi noted. Segmental double contours and ischemic wrinkling of glomerular basement membranes were present. Mild tubular atrophy and interstitial fibrosis were noted. Immunofluorescence revealed mild mesangial C3 and trace IgM staining, with other immune reactants negative. Electron microscopy showed subendothelial lucencies, endothelial fenestration loss, and moderate podocyte effacement. Findings supported arteriolar-predominant TMA without clear immune complex-mediated glomerulonephritis (Figures 2-3). She also underwent genetic testing, which identified a heterozygous pathogenic splice-site variant in CFH (NM_000186.4 c.58+1G > T), consistent with autosomal dominant aHUS.

**Figure 4:**
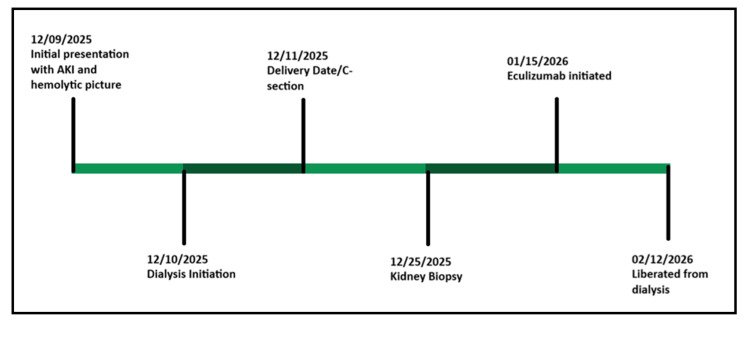
Timeline from presentation until renal recovery, showing delayed initiation of C5 complement inhibitor

Eculizumab therapy was initiated approximately five weeks after initial presentation, following appropriate vaccination and antibiotic prophylaxis. The patient later transitioned to ravulizumab. Approximately four weeks after initiation of complement blockade, her urine output gradually improved, and she was able to achieve dialysis independence. Her most recent creatinine was 2.3 mg/dL, and hemolysis parameters have all recovered (Figure 4).

## Discussion

Pregnancy-associated aHUS accounts for a significant proportion of complement-mediated TMA in women of childbearing age [3-5]. The third trimester and postpartum period represent high-risk windows due to physiologic complement activation. In contrast to HELLP syndrome, complement-mediated TMA often presents with severe kidney failure and limited hepatic involvement [5]. The CFH mutations are among the most common genetic abnormalities in aHUS [2,4]. Heterozygous variants demonstrate reduced penetrance and require environmental triggers such as pregnancy. Splice-site variants may impair synthesis of functional CFH protein, resulting in uncontrolled complement activation on endothelial surfaces.

Distinguishing pregnancy-associated aHUS from other pregnancy-related TMAs such as preeclampsia and HELLP syndrome remains one of the most important clinical challenges in obstetric nephrology. While HELLP syndrome typically improves rapidly following delivery, complement-mediated TMA frequently persists or worsens postpartum [5,6]. Severe kidney injury disproportionate to hepatic involvement, persistent thrombocytopenia or hemolysis after delivery, and dialysis dependence should prompt consideration of aHUS and evaluation for complement dysregulation. In many cases, however, the diagnosis remains uncertain at initial presentation because clinical features overlap and definitive genetic testing may not be immediately available. As a result, delays in targeted therapy are not uncommon in real-world clinical practice.

Eculizumab has transformed prognosis in aHUS, reducing progression to end-stage kidney disease [6]. Early initiation is associated with better outcomes; however, delayed therapy can still result in kidney function recovery [8-10]. In our case, complement inhibition began five weeks after dialysis initiation, yet the patient was able to achieve dialysis independence. This underscores that dialysis dependence should not preclude initiation of complement blockade. The timeline in this case highlights several important principles: severe AKI with normal liver enzymes in pregnancy warrants evaluation for complement-mediated TMA, delivery alone may not reverse kidney failure, kidney biopsy is valuable in distinguishing etiologies, and delayed C5 inhibition can still result in meaningful kidney recovery.

Some reports have demonstrated that patients with aHUS who require dialysis at presentation may still experience renal recovery following complement blockade, particularly when irreversible cortical necrosis is absent [8,11]. Thrombotic microangiopathy primarily affects the microvascular endothelium, and complement inhibition can halt ongoing injury and allow gradual endothelial repair. Our patient’s biopsy demonstrated arteriolar-predominant TMA with relatively limited chronic scarring, which may have contributed to her potential for renal recovery despite dialysis dependence. These findings highlight the importance of kidney biopsy in selected cases where the diagnosis remains uncertain or where prognostic information may influence treatment decisions.

## Conclusions

This case emphasizes the importance of maintaining a high index of suspicion for complement-mediated TMA in pregnant patients with severe kidney dysfunction. Delayed diagnosis may occur due to overlapping obstetric syndromes, logistical barriers to genetic testing, or delays in access to complement inhibitors. However, accumulating evidence suggests that complement blockade should still be considered even when treatment cannot be initiated immediately. Our case adds to the growing literature demonstrating that meaningful renal recovery remains possible despite delayed initiation of therapy, reinforcing the need for continued diagnostic evaluation and therapeutic intervention even after the acute obstetric event has resolved.
